# Computerized Cognitive Behavioral Therapy for Anxiety and Depression in Farming Communities: Mixed Methods Feasibility Study of Participant Use and Acceptability

**DOI:** 10.2196/42573

**Published:** 2023-06-19

**Authors:** Harriet L Bowyer, Ruth Pegler, Christopher Williams

**Affiliations:** 1 Department of Psychology Glasgow Caledonian University Glasgow United Kingdom; 2 Paediatric Clinical Psychology Service National Health Service Scotland Greater Glasgow & Clyde Glasgow United Kingdom; 3 School of Health & Wellbeing University of Glasgow Glasgow United Kingdom

**Keywords:** computerized cognitive behavioral therapy, cCBT, cognitive behavioral therapy, CBT, farmer, depression

## Abstract

**Background:**

Farmers have higher rates of depression than nonfarmers and higher rates of suicide than the general population. Several barriers to help seeking have been identified in farmers, which may be overcome by offering web-based mental health support. Computerized cognitive behavioral therapy (cCBT) is an effective intervention used to prevent and treat mild to moderate depression but has not been evaluated in the farming community.

**Objective:**

This study explored the feasibility of delivering a cCBT course tailored to farmers using a mixed methods approach.

**Methods:**

Farmers (aged ≥18 years) with no, minimal, or moderately severe depressive symptoms (Patient Health Questionnaire–9 [PHQ-9] score <20) were recruited using web-based and offline advertisements and given access to a cCBT course consisting of 5 core modules and automated and personalized email support. Depression (PHQ-9), anxiety (General Anxiety Disorder–7), and social functioning (Work and Social Adjustment Scale) were measured at baseline and the 8-week follow-up. Wilcoxon signed rank tests assessed changes in scores for all outcome measures over time. Telephone interviews focusing on participant use and satisfaction with the course were analyzed using thematic analysis.

**Results:**

Overall, 56 participants were recruited; 27 (48%) through social media. Overall, 62% (35/56) of participants logged into the course. At baseline, almost half of the participants reported experiencing minimal depressive symptoms (25/56, 45%) and mild anxiety (25/56, 45%), and just over half (30/56, 54%) reported mild to moderate functional impairment. Posttreatment data were available for 27% (15/56) of participants (41/56, 73% attrition rate). On average, participants experienced fewer depressive symptoms (*P*=.38) and less functional impairment (*P*=.26) at the 8-week follow-up; these results were not statistically significant. Participants experienced significantly fewer symptoms of anxiety at the 8-week follow-up (*P*=.02). Most participants (13/14, 93%) found the course helpful and easy to access (10/13, 77%) and the email support helpful (12/14, 86%). Qualitative interviews identified heavy workloads and mental health stigma within the farming community as barriers to help seeking. Participants thought that web-based support would be helpful, being convenient and anonymous. There were concerns that older farmers and those with limited internet connections may have difficulty accessing the course. Improvements regarding the layout and content of the course were suggested. Dedicated support from someone with farming knowledge was recommended to improve retention.

**Conclusions:**

cCBT may be a convenient way of supporting mental health within farming communities. However, challenges in recruiting and retaining farmers may indicate that cCBT supported only by email may not be an acceptable mode of mental health care delivery for many; however, it was valued by respondents. Involving farming organizations in planning, recruitment, and support may address these issues. Mental health awareness campaigns targeting farming communities may also help reduce stigma and improve recruitment and retention.

## Introduction

### Background

The most recent data estimate that there are approximately 101,900 farmers in the United Kingdom [1], with the farming industry playing a substantial role in the British economy, earning £5998 million (US $7431.76 million) in 2021 [2]. In the United States, high rates of suicide have been reported among farmers, ranchers, and other agricultural managers, with a risk statistically higher than the population rate [3]. This was supported by a systematic review and meta-analysis published in 2018, which highlighted that workers in the agricultural, forestry, and fishery industries are at increased risk of suicide [4]. Studies have also indicated that full-time male and female farmers have higher levels of depression and anxiety than nonfarmers, with odds ratios of 2.3 and 2.1, respectively [5], and that female farmers report even higher levels of anxiety, stress, and depression than their male counterparts [6,7]. These mental health difficulties have a considerable personal, social, and economic cost [8].

A recent systematic review identified a variety of stressors that may place farmers at increased risk of mental health difficulties, such as financial concerns, inconsistent weather, exposure to pesticides, and poor physical health or injury [9]. Research has also indicated that social, cultural, and geographical isolation may play a substantial role in farmers’ mental health [10,11]. It is likely that these stressors (eg, work-based stressors and isolation) not only place farmers at increased risk of mental health difficulties but that their mental health will likely have an impact on their functioning. For example, research has indicated that there is an association with functional impairment (such as social and daily activities) in those who experience even low levels of depression [12]. Unfortunately, research has highlighted several barriers to help seeking in farmers: having limited knowledge about and poor recognition of mental health difficulties [13], being reluctant to admit to experiencing mental health difficulties [14], and having substantial demands at work and poor access to physical and mental health services [15]. A recent qualitative study that focused on barriers to and facilitators of mental health help seeking in farmers indicated the importance of health care professionals being available and accessible, having farming knowledge, and offering continuity of care [16].

A way of tackling barriers to help seeking may be to offer farmers web-based mental health support. There is an increasing evidence base for computerized cognitive behavioral therapy (cCBT) [17], with cCBT recommended by the National Institute for Health and Care Excellence for treating mild to moderate depression [18]. A systematic review indicated that cCBT may be more acceptable to individuals living in rural versus urban communities; that rural communities are less likely to want face-to-face contact with mental health professionals; and that cCBT may help reduce concerns regarding visibility and, therefore, confidentiality when help seeking [19]. However, it is recognized that providing support in this way may bring about additional barriers for some because of poor connectivity in rural areas [16].

### Objectives

Organizations such as the Farming Community Network and the Royal Agricultural Benevolent Institute provide farmers with support via email and telephone helplines, and in 2022, the Royal Agricultural Benevolent Institute launched a counseling service for farmers to access face-to-face, by telephone, or by videoconferencing [20], which would benefit from evaluation once established. A recent systematic review failed to find any evidence for direct clinical interventions for farmers, suggesting that there is an urgent need for research in this area [21]. The authors also highlighted the importance of engaging farmers in the development of interventions as they are more likely to be acceptable and effective for this population. Given the limited evidence base on specific interventions aimed at preventing and supporting farmers’ mental health, the evidence base for cCBT, and its potential to overcome a number of barriers to help seeking, this study aimed to assess the feasibility of offering farmers a cCBT-based life skills course. In line with the objectives of a feasibility study outlined by Orsmond and Cohn [22], this feasibility study aimed to test the ability to recruit; gather baseline and follow-up data; test the delivery and acceptability of the intervention; and gain an estimate of uptake, dropout, and treatment effect to inform a sample size for a future definitive randomized controlled trial (RCT).

## Methods

### Design

This was a feasibility study using a mixed methods design: a single-arm repeated-measure design and one-to-one semistructured interviews.

### Participants

Eligible participants were members of the farming community aged ≥18 years residing in the United Kingdom. The term *farmer* was operationalized using the European Regulations (1307/2013) definition [23]: “A natural or a legal person (or a group of natural or legal persons) whose holding (production units) is situated within the territory of the European Union, and who exercises an agricultural activity*.*” It was planned that all participants would have mild to moderate symptoms of depression (as indicated by a score of between 5 and 19 on the Patient Health Questionnaire–9 [PHQ-9]) [24] at baseline; however, because of difficulties recruiting, this was amended to allow individuals with any score of <20 on the PHQ-9 to take part. The rationale for this was related to evidence suggesting that cCBT can be used as an effective intervention to prevent depression, which is likely of particular importance given the increased risk of mental health difficulties within the farming community [25]. Furthermore, research suggests that the current thresholds used for the PHQ-9 may result in depression remaining undetected in many individuals [26]; therefore, including participants with a score of <5 may have improved sensitivity. Individuals were excluded from the study if they (1) did not complete a consent form, (2) did not provide their general practitioner’s contact details, (3) were considered to have severe depression (as indicated by ≥20 PHQ-9 score), (4) consumed more than double the weekly recommended alcohol limits at the time (men: >50 units of alcohol per week; women: >35 units of alcohol per week [27]), or (5) were currently receiving psychological treatment*.*

Participants were recruited between November 2016 and March 2017 using a variety of recruitment methods. The Royal Scottish Agricultural Benevolent Institute (RSABI) and the National Farmers Union of Scotland (NFUS) distributed a joint news release. Free one-off advertisements were placed in Country Squire Magazine, Countryman Magazine, Farmland Magazine, Farming Forum, and the Scottish Dairy Hub. Flyers were distributed by the NFUS, RSABI, and Scottish Association of Young Farmers Clubs at agricultural meetings. A radio interview with BBC Radio Scotland “Out of Doors” was broadcast on November 19, 2016, describing the purpose of the study. Facebook and Twitter pages were also created to promote the study. A marketing company was used to target farmers on Facebook, whereas all promotion on Twitter was conducted by the main researcher (HB). All advertisements directed participants to a web-based recruitment site containing a participant information sheet and a link to the consent form and baseline questionnaire hosted by SurveyMonkey (Momentive Global Inc). The baseline questionnaire asked participants how they had heard about the study.

### Measures

The primary outcome measures were to describe the demographic characteristics of the sample, assess the ability to recruit participants for the study, and assess the ability to gather outcome measures at baseline and follow-up. Demographic data were obtained using a questionnaire developed by the research team.

Secondary measures included self-reported (1) depression (the likely primary outcome in any future substantive study) using the PHQ-9 (a total of 9 items on a 4-point scale: “not at all” to “nearly every day” scored from 0 to 3), which classifies depression into 5 categories: minimal depression (score range 0-4), mild depression (score range 5-9), moderate depression (score range 10-14), moderately severe depression (score range 15-19), and severe depression (score range 20-27); (2) anxiety using the General Anxiety Disorder–7 (GAD-7; a total of 7 items on a 4-point scale: “not at all” to “nearly every day” scored from 0 to 3) [28], which classifies anxiety into 4 categories: minimal anxiety (score range 0-4), mild anxiety (score range 5-9), moderate anxiety (score range 10-14), and severe anxiety (score of ≥15); and (3) social functioning using the Work and Social Adjustment Scale (WSAS; a total of 5 items on a 9-point scale: “not at all” to “very severely” scored from 0 to 8) [29], which classifies functional impairment into 3 categories: subclinical (score range 0-9), substantial functional impairment but less severe clinical symptomatology (score range 10-20), and moderately severe or worse psychopathology (score of ≥21).

These measures were chosen because of the high rates of comorbidity between depression and anxiety and related functional impairment [30]. Participants’ satisfaction with the intervention was assessed using the Client Satisfaction Questionnaire–8 (CSQ-8; a total of 8 items on a 4-point scale scored from 1 to 4, with higher scores indicating greater satisfaction) [31] and some additional use and acceptability questions developed by the research team. The PHQ-9 [24], GAD-7 [32], WSAS [29], and CSQ-8 [33] are all reliable and valid measures.

### Procedure

Informed consent was obtained via a web-based survey before completion of the baseline questionnaire. The consent form asked participants to consent to their general practitioner being contacted by the research team if there were concerns about any active risk (ie, if they indicated on their PHQ-9 that they had had thoughts that they would be better off dead or of hurting themselves in some way nearly every day). During the consent process, participants were also offered the opportunity to volunteer to take part in an in-depth semistructured telephone qualitative interview at the end of the study. After providing informed consent, participants completed a baseline questionnaire pack. Individuals who met the inclusion criteria were given immediate access to the “Living Life to the Full for Farming Communities” research website, a modified version of the “Living Life to the Full” (LLTTF) web-based course [34] based at a new URL for the purposes of the evaluation.

The course consisted of 5 core modules, with content rewritten to focus on farming issues. These modules aimed to teach life skills to support individuals using a cognitive behavioral therapy (CBT) framework. The core modules included understanding your feelings (self-formulation), doing things that make you feel better (behavioral activation), looking at things differently (negative and anxious thinking), how to fix almost everything (problem-solving), and tension control training (relaxation). Participants were encouraged via the site and email support to begin with the first module, “understanding your feelings.” On the farming-specific research website, these core modules included examples that were modified to be relevant and of interest to the farming community informed by unpublished observations by a previous researcher (Mr Ross Lamont) and advice provided by the RSABI and NFUS. Additional optional modules aimed to support more specific difficulties such as how to reduce alcohol consumption, stop smoking, or improve sleep. These are optional modules available for all LLTTF packages, with research linking alcohol use, smoking, and insomnia to depression and anxiety [35-37]. Each module consisted of a slideshow presentation guided by audio and access to linked downloadable worksheets and web-based books. Individuals could choose to work through all the modules in sequence in their own preferred order or could just choose the modules that were most relevant to them. When working through the course, participants received weekly, standardized, and automated support emails plus weekly short support emails using a standard structure that was “personalized” to a small extent from an independent supporter (a trainee clinical psychologist) for 8 weeks to encourage engagement with and completion of the course modules. The independent supporter could log onto the web-based course and see which modules the participant was working through or had completed that week to inform the email content. A standardized email support template was used and modified to include responses to participant queries and highlight individual progress made on the course that week (Multimedia Appendix 1). This support aimed to motivate participants and encourage site use.

After 8 weeks, participants were emailed a web link so that they could recomplete the baseline measures with additional questions regarding use of and satisfaction with the web-based course. If participants did not log onto the website or did not complete the follow-up measures, they were sent up to 2 reminder emails. If they did not respond to these emails, they were regarded as lost to follow-up. Where possible, reasons for withdrawal from the study were gathered to inform future research.

Those who consented to take part in a telephone interview were also contacted after 8 weeks and interviewed until data saturation was reached. The telephone interviews were semistructured and aimed to gather information on what participants thought of the web-based course and any recommendations for future use. The interview schedule was informed by an unpublished research study conducted with farmers by a previous researcher (Mr Ross Lamont) and covered questions regarding acceptability of the course; what they thought of the web-based setting, course content, and support offered; whether they learned and used any skills; and specific questions about the research design (eg, the length and content of questionnaires, communication with the research team, and suggested changes for future research). The interview schedule was tested with a peer (a trainee clinical psychologist) to ensure that the questions were clear and accessible. The interviews were digitally recorded, transcribed, and anonymized. Any participant identifiers were removed and placed in a separate password-protected file and saved on an encrypted device to allow participants to withdraw their data should they request them. Participants were not compensated for taking part in the quantitative study; however, participants who took part in the qualitative interviews received a £5 (US $6.20) Amazon voucher to compensate them for their time.

### Data Analysis

Descriptive statistics were used to describe the sample. Exploratory statistics such as chi-square tests and independent 2-tailed *t* tests were used to determine any baseline differences between participants who did and did not log onto the course. Tests of normality were carried out on the change in scores between outcome measures at baseline and the 8-week follow-up. Shapiro-Wilk tests, distribution histograms, *Q*-*Q* plots, and box plots suggested that there was a substantial difference between the data distribution and the normal distribution; therefore, nonparametric statistics were used. Wilcoxon signed rank tests assessed changes in scores for all secondary outcome measures over time. A power calculation for a future study is reported. Quantitative analyses were performed using SPSS (version 21; IBM Corp) [38]. The audio recorded interviews were transcribed and then anonymized. Qualitative data analysis was conducted using thematic analysis [39], which aims to identify common themes or patterns within the data by following a 6-phase framework (data familiarization, generating initial codes, searching for themes, reviewing themes, defining themes, and writing up). NVivo (version 11; QSR International) [40] was used for data management and interpretation. Initial themes were identified by HB and discussed and confirmed with RP.

### Ethics Approval

Ethics approval was obtained from the University of Glasgow Medical and Veterinary and Life Sciences ethics panel (reference 200160003; approval date: August 31, 2016).

## Results

### Recruitment Methods

**Figure 1:**
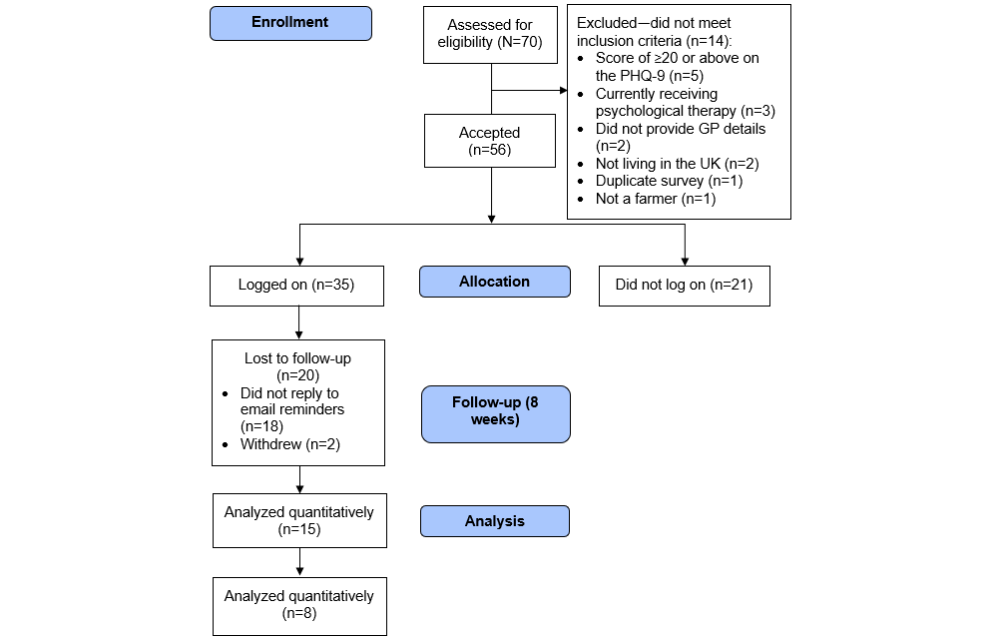
Participant flowchart. GP: general practitioner; PHQ-9: Patient Health Questionnaire–9; UK: United Kingdom.

A total of 70 individuals consented to participate and completed the baseline questionnaire; 56 (80%) participants met the inclusion criteria (Figure 1).

Just under half (27/56, 48%) of the participants were recruited via social media (Table 1). Almost half (23/56, 41%) were recruited using a free Twitter account, with minimal numbers (4/56, 7%) recruited using a paid-for advertisement on Facebook. A post placed on the Farming Forum recruited 32% (18/56) of the participants. The NFUS and RSABI press release and advertisements placed on the web or in paper-based magazines and newspapers appeared to be less successful in recruiting participants; the radio interview appeared to be ineffective in recruiting participants.

**Table 1:** Recruitment methods (n=56).

	Participants, n (%)
Social media	27 (48)
The Farming Forum	18 (32)
National Farmers Union of Scotland	3 (5)
Farming magazines or newspapers	2 (4)
Farming community network	1 (2)
A farming website	1 (2)
Word of mouth	4 (7)

### Sample Characteristics

Participants (n=56) were recruited from across the United Kingdom: 63% (22/35) were English, 23% (8/35) were Scottish, and 14% (5/35) were Welsh. All participants were of White ethnicity. Most participants were male (43/56, 77%), aged between 35 and 54 years (35/56, 62%), and married or living with their partner (44/56, 79%; Table 2). Before entering the study, most participants (47/56, 84%) had been using the internet for ≥7 years. Most participants (33/56, 59%) had experienced mental health difficulties in the past, with 73% (24/33) of them having received mental health support.

Owing to a technical error, baseline data on 1 item of the PHQ-9 were missing for 5% (3/56) of the participants. Following advice from a statistician, missing data were dealt with using mean imputation, which is supported by the existing literature using the PHQ-9 [41]. At baseline, participants had a median PHQ-9 score of 7 (IQR 4-11; minimal symptoms), a median GAD-7 score of 6 (IQR 2-9; mild symptoms), and a median WSAS score of 9 (IQR 4-12; mild to moderate functional impairment). Baseline scores on the PHQ-9 indicated that 27% (15/56) fell within the normal range, 45% (25/56) had minimal depressive symptoms, 21% (12/56) had mild depressive symptoms, and 7% (4/56) had moderate depressive symptoms. Baseline scores on the GAD-7 indicated that 32% (18/56) fell within the normal range, 45% (25/56) had mild anxiety, 18% (10/56) had moderate anxiety, and 5% (3/56) had severe anxiety. Baseline scores on the WSAS indicated that a total of 23% (13/56) fell within the subclinical range, 54% (30/56) had mild to moderate functional impairment, and 23% (13/56) had moderate to severe functional impairment.

**Table 2:** Demographic characteristics of the sample (n=56).

			Total, n (%)^a^		Logged on				*P* value^b^ (chi-square test)
					Yes (n=35), n (%)		No (n=21), n (%)		
**Age group (years)**									
	18-24	1 (2)		1 (3)		—^c^		—	
	25-34	6 (11)		4 (11)		2 (10)		—	
	35-44	16 (29)		11 (31)		5 (24)		—	
	45-54	19 (34)		10 (29)		9 (43)		—	
	55-64	12 (21)		7 (20)		5 (24)		—	
	≥65	2 (4)		2 (6)		—		—	
**Sex**									.33
	Male	43 (77)		25 (71)		18 (86)			
	Female	13 (23)		10 (29)		3 (14)			
**Marital status**									
	Single	6 (11)		3 (9)		3 (14)		—	
	Married or living with partner	44 (79)		27 (77)		17 (81)		—	
	Separated or divorced	5 (9)		4 (11)		1 (5)		—	
	Widowed	1 (2)		1 (3)		—		—	
**Religion**									.28
	Christian	44 (79)		25 (76)^d^		19 (90)			
	Atheist or agnostic	10 (18)		8 (24)^d^		2 (10)			
**Main type of business**									
	Beef	10 (18)		6 (17)		4 (19)		—	
	Combinable crops	15 (27)		8 (23)		7 (33)		—	
	Dairy	5 (9)		4 (11)		1 (5)		—	
	Pigs	3 (5)		2 (6)		1 (5)		—	
	Potatoes	3 (5)		2 (6)		1 (5)		—	
	Poultry	1 (2)		1 (3)		—		—	
	Sheep	13 (23)		9 (26)		4 (19)		—	
	Mixed farming	6 (11)		3 (9)		3 (14)		—	
**Time spent on the farm**									.12
	<4 hours per day	6 (11)		5 (14)		1 (5)			
	4-6 hours per day	3 (5)		1 (3)		2 (10)			
	6.5-8 hours per day	10 (18)		7 (20)		3 (14)			
	8.5-10 hours per day	15 (27)		12 (34)		3 (14)			
	10.5-12 hours per day	14 (25)		5 (14)		9 (43)			
	≥12 hours per day	8 (14)		5 (14)		3 (14)			
**Past mental health problems**									.44
	Yes	33 (59)		22 (63)		11 (52)			
	No	23 (41)		13 (37)		10 (48)			
**Currently taking medication for mental health**									.08
	Yes	14 (25)		6 (17)		8 (38)			
	No	42 (75)		29 (83)		13 (62)			
**How long have you been using the internet (years)?**									
	1-3	2 (4)		—		2 (10)		—	
	4-6	7 (12)		5 (14)		2 (10)		—	
	≥7	47 (84)		30 (86)		17 (81)		—	

^a^The n value varies because of missing data.

^b^Fisher exact test used for cell counts of <5.

^c^No test of significance was applicable for variables with cell counts of <1.

^d^n=33.

### Primary Outcome Measures

#### Attrition and Adherence

Posttreatment data were collected from 27% (15/56) of the participants who were eligible to take part in the study—an attrition rate of 73% (41/56). A total of 62% (35/56) of the participants logged onto the website. In total, 4% (2/56) of the participants withdrew from the study after logging in. A total of 2% (1/56) of the participants did not have enough time to give to the course, and 2% (1/56) of the participants withdrew because they felt that their low mood affected their ability to engage with the course. Exploratory analyses were conducted on demographic characteristics with cell counts of ≥1 between those who did and did not log onto the course and those who did and did not complete the follow-up measures. No significant differences were found with regard to sex (*P*=.33), religion (*P*=.28), time spent on the farm (*P*=.12), past mental health problems (*P*=.44), and current use of medication for their mental health (*P*=.08). There were no significant differences between those who did and did not log onto the course on all baseline outcome measures (all *P*>.05; Table 3) and those who did and did not complete the follow-up questionnaire (Multimedia Appendix 2).

Of those who logged onto the course, a total of 57% (20/35) started the course (defined as those who started any of the modules in no particular order), and 14% (5/35) of them completed all 5 core modules within 8 weeks. Of those who started the course, the average number of completed modules was 1.76 (SD 1.97; Table 4). Reasons why participants did not complete the course were not available.

**Table 3:** Participants’ scores on the secondary outcome measures at baseline (N=56).

	Total sample, median (IQR)	Logged on (n=35), median (IQR)	Did not log on (n=21), median (IQR)	*P* value
PHQ-9^a^	7.0 (4-11)	7.0 (5-10)	6.8 (1-12)	.44
GAD-7^b^	6.0 (2-9)	6.0 (4-9)	6.0 (2-11)	.70
WSAS^c^	9.0 (4-12)	12.0 (8-16)	12.0 (7-23)	.65

^a^PHQ-9: Patient Health Questionnaire–9.

^b^GAD-7: General Anxiety Disorder–7.

^c^WSAS: Work and Social Adjustment Scale.

**Table 4:** Acceptability and use of the web-based course for farmers in the total sample (n=35)^a^.

Variable		Values
**Started the course, n (%)**		20 (57)
	“Understanding your feelings” completed, n (%)	13 (65)
	“Doing things that make you feel better” completed, n (%)	7 (35)
	“Looking at things differently” completed, n (%)	5 (25)
	“How to fix almost everything” completed, n (%)	6 (30)
	“Tension control training” completed, n (%)	6 (30)
	Completed all modules, n (%)	5 (25)
	Modules completed (n=20), mean (SD; range)	1.76 (1.97; 0-5)
e-Books read, mean (SD; range)		1.14 (1.50; 0-5)
Number of log-ins, mean (SD; range)		3.20 (3.53; 1-15)

^a^All modules were unlocked, so participants did not need to work through the course in a sequential manner.

#### Email Contact per Participant

Participants received a total of 8 automatic weekly emails and a mean of 5.86 (SD 0.77; range 3-7) personalized support emails. Participants sent a mean of 0.94 (SD 1.28; range 0-5) emails in response to the personalized emails.

### Secondary Outcome Measures

#### Therapeutic Change

This exploratory feasibility study was intentionally not powered to detect differences in scores over time and, instead, aimed to record uptake and delivery and provide an estimate of the intervention effect. However, exploratory analyses were conducted to observe any trends in the data. On average, participants experienced fewer depressive symptoms at the 8-week follow-up (median 7, IQR 4-9; 15/56, 27%) than at baseline (median 8, IQR 4-12; 15/56, 27%); this was not significant (*z*=−0.83; *P*=.38). Overall, participants experienced less anxiety at the 8-week follow-up (median 4, IQR 2-11; 15/56, 27%) than at baseline (median 6, IQR 2-13; 15/56, 27%); this difference was significant (*z*=−2.28; *P*=.02). On average, participants experienced less functional impairment at the 8-week follow-up (median 9, IQR 4-12; 15/56, 27%) than at baseline (median 9, IQR 4-14; 15/56, 27%); this difference was not significant (*z*=−1.12; *P*=.26; Table 5). A sensitivity analysis including only participants with baseline PHQ-9 scores of ≥5 found no change in the significance of the results (Multimedia Appendix 3).

**Table 5:** Change in secondary outcome measures over time.

	Baseline, median (IQR)	Follow-up, median (IQR)	*P* value
PHQ-9^a^	8 (4-12)	7 (4-9)	.38
GAD-7^b^	6 (2-13)	4 (2-11)	.02
WSAS^c^	9 (4-14)	9 (4-12)	.26

^a^PHQ-9: Patient Health Questionnaire–9.

^b^GAD-7: General Anxiety Disorder–7.

^c^WSAS: Work and Social Adjustment Scale.

#### Sample Size Calculation

The PHQ-9 change score enabled a sample size calculation to be completed to determine the number of participants that would be needed for a future RCT using a waitlist control. On the basis of the assumption that there will be no change in PHQ-9 scores in the control group (mean PHQ-9 score change 0) and the same change in PHQ-9 scores observed in this study in the intervention group (mean PHQ-9 score change 1.47) and that there will be a similar SD between the groups, as observed in this study (SD 5), a sample size of 352 participants with baseline and follow-up data would be needed to have 80% power to detect differences where *P*<.05. In this study, follow-up data were available for 27% (15/56) of the participants. To allow for an attrition rate of 73% in a future study, a sample size of 1303 participants would be required. The calculation also assumes that those recruited have the same low levels of initial depression and poor retention as those of the current group. It is likely that the intervention effect would be larger if a more initially depressed group could be recruited, and the sample size required would benefit substantially by reducing dropout.

#### Participant Satisfaction

Overall, participants who completed the 8-week follow-up questionnaire reported a medium to high level of satisfaction with the web-based course. The mean score on the CSQ-8 was 20.87 (SD 0.64; range 20-22; 15/56, 27%). There were some missing data on some of the additional use and acceptability questions developed by the research team. However, most participants reported finding the web-based course helpful (13/14, 93%) and easy to access (10/13, 77%). A total of 53% (8/15) felt able to perform the activities suggested by the course. Most participants (12/14, 86%) found the email support helpful (Table 6).

**Table 6:** Responses to the additional use and acceptability questions designed by the research team.

Satisfaction questions	Strongly disagree, n (%)	Slightly disagree, n (%)	Neither agree nor disagree, n (%)	Slightly agree, n (%)	Strongly agree, n (%)
I found the course helpful (n=14)^a^	1 (7)	N/A^b^	N/A	7 (50)	6 (43)
I found the course easy to access (n=13)^a^	N/A	1 (8)	3 (23)	2 (15)	8 (62)
I was able to do the activities suggested by the course (n=15)^a^	1 (7)	N/A	5 (33)	5 (33)	3 (20)
I found the email support helpful (n=14)^a^	N/A	N/A	3 (21)	4 (29)	8 (57)

^a^The n value varies because of missing data.

^b^N/A: not applicable.

### Telephone Interviews

#### Overview

A total of 14% (8/56) of the participants took part in a qualitative telephone interview (Table 7). The participants were from England (5/8, 62%), Scotland (2/8, 25%), and Wales (1/8, 12%). The participants varied in terms of the type of farming they engaged in: dairy (3/8, 38%), beef (1/8, 12%), combinable crops (1/8, 12%), pigs (1/8, 12%), potatoes (1/8, 12%), and sheep (1/8, 12%).

**Table 7:** Description of participants in the telephone interviews.

Participant number	Sex	Age group (years)	Modules completed (n=5), n (%)
1	Female	35-44	5 (100)
2	Male	55-64	5 (100)
3	Male	45-54	5 (100)
4	Female	≥65	0 (0)
5	Male	35-44	5 (100)
6	Female	25-34	2 (40)
7	Female	25-34	0 (0)
8	Male	55-64	5 (100)

In total, 4 broad themes were identified using thematic analysis: barriers to help seeking, delivery of mental health support, usability of the web-based course for farming communities, and the content of LLTTF for farming communities.

#### Barriers to Help Seeking

Participants discussed the difficulties in seeking help for mental health difficulties within the farming community. Mental health stigma was discussed, with participants describing farmers as proud and stoic:

I think the biggest problem is going to be with farmers is that they’re quite sort of independent people and they don’t like to admit their short comings...some people feel that sort of weakness and that, until it’s appreciated that a lot of people are suffering from it, you won’t get much further forward.

Participant 2

Get on with it, there’s nothing wrong with you, get on with it. That sort of, that attitude and that is going to take an awful lot of shifting.

Participant 4

Participants also discussed the difficulty in finding the time to engage with mental health support because of work commitments:

The seasons are constantly chasing you and if the suns shining that day well, you’ve got to go and make the hay...or if your sheep start lambing, well you’ve got to go and help your sheep. I think the hardest bit about farming, you just can’t pack it up at 5 o’clock and go away. You have your busy times and your quiet times so it would be trying to capture farmers in some of the quieter months.

Participant 1

Some participants reported that, even when they engaged with mental health support, it could be difficult to find the time to try out some of the recommended strategies:

All through the summer when you’re busy harvesting that’s a job to get exercise really.

Participant 5

#### Delivery of Mental Health Support

Overall, participants were positive about receiving mental health support on the web. They frequently discussed how the convenience of web-based support overcame the difficulty of finding time to seek help:

Being online at least it can be done at any time or day at night. When you have them [the modules] only being short as well...you can sit down, do that module very quickly...that’s great is that. Whereas if someone was to go see someone...you’re never clean enough to go somewhere, go out, come back. It’s nearly half a day’s work whereas ten minutes at end of day or half an hour at end of day, you can quite easily do that. It’s something you say, well alright I can do that but finding an appointment to go and see a doctor is difficult enough.

Participant 8

The importance of anonymity when seeking help was also discussed:

I think being discreet is the biggest thing. It’s something you can do without anyone if you don’t feel like you want anyone else to know.

Participant 7

Participants did report a number of concerns regarding receiving mental health support on the web. First, there were concerns that it would not be accessible to older farmers:

I think generally for farmers online is better. If they’re the right you know...in terms of what age group you’re talking to because obviously the older the farmer than maybe they’re not gonna be quite so engaged with technology...whereas the younger ones probably are.

Participant 3

Participants also reported that, because of rural locations, a lot of farmers have difficulties with their internet connection:

The only thing is internet connection because some people struggle still with internet connection in rural areas. That would be my only concern.

Participant 7

#### Usability of the Web-Based Course for Farming Communities

Participants described finding the web-based CBT package easy to use. They reported that the modules were the right length, and several participants commented on the usefulness of it being an unlocked design where participants could pick and choose modules they wanted to complete:

They [the modules] were just a nice length of time. They were short enough to be able to go in and do one course and come back out. You could dip in and dip out. I think if they were any longer it would have put you off...and you didn’t have to do them in any order you could do whichever ones you chose sort of when.

Participant 1

In terms of suggested improvements that could be made to the course, a number of participants reported that the layout of the website could be clearer:

The only thing when I first logged in and I looked at the screen and I saw like the three boxes at the top and I thought “oh ok, right, which one do I click first?” It wasn’t just 101% clear...where do I click just to get me started?

Participant 1

Participants wanted to be able to download some of the modules because of difficulties with their internet connection:

The only thing I would have liked would be able to download the handbooks for each module so you can look at them at your leisure rather than being on the internet, being stuck in the countryside with low internet speed is a bit of a pain.

Participant 2

Participants also described wanting the flexibility to be able to access the e-books and worksheets in a variety of formats, such as print or editable PDFs, and on a variety of technological devices.

#### Content of the Web-Based Course for Farming Communities

Participants found it helpful to learn how to deal with negative thoughts, problem-solving, and self-care:

The things about negative thoughts, don’t let negative thoughts get you down. That was quite an important one to me as well.

Participant 1

It sounds obvious from when you watch the slides and then you come away and you think “well actually I can do that, I can break it down into smaller chunks and deal with a little bit of a problem at a time and it will help” and half the problem goes away because you realise that you can deal with it.

Participant 6

The You-time ones [modules about self-care], taking time to yourself. Not really things I would have thought about, sort of being a farmer you put yourself to the back of the line so it’s all of these things you don’t really think about, any of these aspects um so see it written down is really simple.

Participant 7

Participants reported finding the email support useful, particularly as a motivator to continue accessing the course:

I thought it was very good and...but for that I wouldn’t have tried for as long as I had.

Participant 4

Email was the preferred method of support because of its convenience:

Again it’s sort of a you can pick it up and put it down as you choose...you don’t have to stop what you’re doing in the middle of the day to sort out an email do you?

Participant 6

Participants were happy with the frequency of the support emails (weekly).

## Discussion

### Principal Findings

The aim of this study was to assess the feasibility of offering a web-based CBT-based life skills course to members of the farming community. To the authors’ knowledge, this is the first study of its kind, providing important information on how to engage members of the farming community with mental health support.

Web-based advertising targeted toward the farming community was the most successful recruitment method. A recent systematic review indicated that social media can be an effective method for recruiting individuals from hard-to-reach populations for medical research [42]; however, it is unclear how representative a web-based sample is. Research has highlighted that, in 2016, just >60% of adults in the United Kingdom were using social networking sites and that only 23% of those aged ≥65 years used them [43]. Only 4% (2/56) of the sample in this study were aged ≥65 years; therefore, older people were likely to be underrepresented. It would be important to directly consult older people within the farming community regarding recruitment methods to ensure successful and representative recruitment in the future.

Recruitment related to symptomatology was challenging, and we failed to engage many people with moderate levels of depression. This, coupled with low follow-up rates, meant that the scope for improvement in mood was limited. Qualitative findings highlighted that mental health stigma within the farming community may still act as a barrier to help seeking, a finding supported by the literature [14]. This is also further supported by the fact that most of the recruited sample (33/56, 59%) had received mental health support in the past, indicating that the recruitment strategy was not as effective in reaching those who had never sought mental health support before.

In recent years, a number of mental health awareness campaigns have been run by members of the farming community, such as the Scottish Association of Young Farmers Clubs “Are Ewe OKAY?” campaign [44], the Farming Community Network “Are you really as tough as old boots?” campaign [45], and the Farming Safety Foundation “Mind Your Head” campaign [46]. Therefore, the numbers recruited without depression and those recruited via Twitter may indicate a desire for the community to raise awareness of and prevent mental health difficulties, reduce stigma, and highlight the sources of support available. This could, in turn, improve help seeking in the farming community for those who are symptomatic. Therefore, it may be beneficial for future studies to focus more on improving mental health literacy and the prevention of common mental health difficulties (such as depression and anxiety) in both those who do and do not meet the PHQ-9 threshold for depression, with a short- and longer-term PHQ-9 follow-up for both groups.

Attrition was higher in this study (41/56, 73%) compared with research on some other web-based courses with nonfarming populations (range 26%-27%) [47,48]. Furthermore, adherence to the course was low, with only 9% (5/56) of the participants completing all the core modules and just over half (8/15, 53%) reporting that they could complete the activities suggested by the course.

Although reasons for dropout were not available from participants in this study, the qualitative findings indicated that, although some liked the convenience of working in this way and the email support, some farmers may find it difficult to engage with mental health support because of highly demanding and unpredictable work commitments, a finding supported by the literature [15]. Our study indicated that, for some, having mental health support on the web and, therefore, accessible at any time of the day helps overcome these barriers and was popular, but further research is needed to identify ways of supporting farmers who cannot or perhaps do not prioritize their mental health. This study recruited a number of participants in the approach to spring, a notoriously busy time for most farmers, which may have also affected attrition and adherence. Indeed, a participant who withdrew from the study did so because of the lack of time. However, because of work-related stress, this may also be the time of the year when farmers would most benefit from support. Discussions with the farming community regarding appropriate times of the year for support may be useful to inform future research and ensure that farmers have the resources to prevent mental health difficulties at particularly busy times of the year. Finally, although all participants recruited for the study had internet access, the qualitative interviews indicated that a number of participants did not have regular internet access because of connectivity difficulties. It may be that, if participants cannot regularly access the course because of poor internet connectivity, they may lose the motivation to log into the site and complete the modules. Having modules that could be downloaded or saved offline (eg, in an app) would be an advantage. We did not ask whether having a printed version of the resources would be helpful; however, providing a printed (book-based) version of the materials might be a useful option for those with issues related to internet access.

Satisfaction with the course was moderate to high among those who completed the follow-up questionnaire, with most participants reporting that the tool was helpful (13/14, 93%) and easy to access (10/13, 77%). The qualitative interviews highlighted the importance of anonymity for some participants, which has been highlighted in previous research [19]. Participants particularly valued the email support, which helped motivate them to adhere to the course. This supports previous research indicating that offering support alongside cCBT can improve patient outcomes [49]. A concern about offering a web-based tool was that it may not be an acceptable delivery method for older people. However, the gap in internet use between younger and older age groups in the United Kingdom appears to be narrowing: internet use for individuals aged 65 to 74 years increased by 31% between 2011 and 2019, with 83% of those surveyed using the internet [50].

Although most of the sample (40/56, 71%) had no or minimal symptoms of depression at baseline, most of the sample had some form of anxiety (mild to moderate: 35/56, 62%) or functional impairment (mild to severe: 43/56, 77%), representing individuals who would be referred to cCBT in the community. A significant and meaningful reduction in anxiety was observed even within this small sample size. A future study may benefit from expanding the inclusion criteria to individuals with both depression and anxiety given the high rates of both found in the farming community [5]. Although not the primary focus of this study, the results indicate that it may be worth conducting a larger, adequately powered study addressing stress and distress in the future using the intervention as it is possible that it may reduce symptoms of anxiety over time.

There are a number of limitations to this study. The small sample size, inclusion of many farmers with only mild symptoms of depression, and absence of a control group make it difficult to know whether the trend regarding treatment effect is a true effect. As this was an unfunded project, a future study with external funding may benefit from recruiting over a longer period using a targeted recruitment strategy on social media platforms. This would hopefully incorporate quieter working periods for certain seasonal farmers. Using largely prestructured or standardized support emails set remotely by a psychology student might not be as effective as having support delivered by someone with a farming background [16]. In a future study using this cCBT resource, the support(s) offered will need to be reviewed and the ability to provide live support (through face-to-face, via live chat, or by telephone) as well as email support options should be explored to try to maximize engagement and retention. Recruitment and support via existing supportive organizations that are well known within farming communities might be a helpful way forward. A future adequately powered RCT targeting those with higher scores on the PHQ-9 or GAD-7 (moderate anxiety or higher) and recruited and supported by personal recommendation (farming organizations or health or community supports) might help determine the likely clinical effect and acceptability of offering cCBT to farmers with more substantial depression or anxiety. Most individuals who took part in the telephone interviews (5/8, 63%) had completed all 5 modules and, as such, may be more likely to report higher levels of satisfaction. Future qualitative studies would benefit from using a sampling frame to ensure that opinions from participants with varied experiences of the web-based course are captured, including those who dropped out or only partially engaged with the intervention, thereby reducing the chance of bias.

### Conclusions

cCBT modified for the farming community may be a convenient way of offering mental health support to a proportion of farmers who face a number of barriers to help seeking. However, difficulties in recruiting and retaining farmers may indicate that internet-based mental health support is not an acceptable mode of delivery for all in the community or may not fully overcome difficulties that inhibit engagement with mental health services; for example, mental health stigma, high workloads, and poor internet connectivity. Retention may be improved by providing access to the course in written form and also by making recommended amendments to the course, for example, allowing the course content to be downloaded and, therefore, available offline. Combining quantitative and qualitative research methods in this study helped gain rich information on the advantages and disadvantages of offering mental health support on the web. A future funded study could address the low adherence rate of this study by increasing the frequency and type of support provided to individuals to motivate them to continue with the intervention, as well as extending the time frame on which this study was conducted to overcome (for some) the work barriers that affected intervention adherence. Given the risk of mental health difficulties, perceived stigma, and reduced help seeking reported in the farming community, it may be of benefit to develop new research questions related to mental health literacy and prevention of depression using a cCBT intervention for the farming community.

## Appendix

Multimedia Appendix 1 Standardized email template to guide email support.

Multimedia Appendix 2 Supplementary analysis for those that did and did not complete the follow-up questionnaires.

Multimedia Appendix 3 Sensitivity analysis of change in measures over time for participants with Patient Health Questionnaire–9 scores of ≥5.

## Abbreviations

CBT: cognitive behavioral therapy
cCBT: computerized cognitive behavioral therapy
CSQ-8: Client Satisfaction Questionnaire–8
GAD-7: General Anxiety Disorder–7
LLTTF: Living Life to the Full
NFUS: National Farmers Union of Scotland
PHQ-9: Patient Health Questionnaire–9
RCT: randomized controlled trial
RSABI: Royal Scottish Agricultural Benevolent Institute
WSAS: Work and Social Adjustment Scale

## Data Availability

Study data are available from the corresponding author upon reasonable request.
