# Supplementary material for: Computerized Cognitive Behavioral Therapy for Anxiety and Depression in Farming Communities: Mixed Methods Feasibility Study of Participant Use and Acceptability
Source: JMIR Form Res. 2023 Jun 19;7:e42573. doi: 10.2196/42573 (PMC10337352; doi:10.2196/42573)
Supplement: Multimedia Appendix 1 [file formative_v7i1e42573_app1.docx]

*Supplementary Material 1: Standardised email template to guide email support*

Hi, my name is XXX, I am a Trainee Clinical Psychologist based at the XXX.

My role is to support and encourage you while you work through the online course. You may find that some tasks are difficult or you lose motivation so I am here to help.

Have you managed to register and log on to the website?

How have you been doing?  Did you find the first module helpful?

It is important to complete one module a week to keep up momentum and improve how you feel. The Planner and Review sheets can be extremely helpful and it is important that you make a clear plan at the end of each module for what you would like to try and achieve in the coming week. There are some instructions on how to use them in the Welcome module. Writing a plan down will help you achieve your goals.

Please do use me as a resource to help you get the most out of the course. I am here to help and all correspondence will be kept confidential within the research project, unless I am seriously concerned about your wellbeing.

I look forward to hearing from you.

Kind regards,

XXX
