# Supplementary material for: Computerized Cognitive Behavioral Therapy for Anxiety and Depression in Farming Communities: Mixed Methods Feasibility Study of Participant Use and Acceptability
Source: JMIR Form Res. 2023 Jun 19;7:e42573. doi: 10.2196/42573 (PMC10337352; doi:10.2196/42573)
Supplement: Multimedia Appendix 2 [file formative_v7i1e42573_app2.docx]

| *Supplementary File 2: Supplementary analysis for those that did and did not complete follow-up questionnaires* | | | | |
| --- | --- | --- | --- | --- |
| *Table 1.* Demographic characteristics of the sample | | | | |
| *Categorical Variables* | *Total*  (n=56; %)^a^ | *Completed follow-up* | | *x^2^*  *P-value****^a^*** |
|  |  | *Yes*  (n=15) % | *No*  (n=41) % |  |
| *Age* |  |  |  |  |
| 18-24 | 1 (1.8) | - | 1 (2.4) |  |
| 25-34 | 6 (10.7) | 3 (20.0) | 3 (7.3) |  |
| 35-44 | 16 (28.6) | 3 (20.0) | 13 (31.7) |  |
| 45-54 | 19 (33.9) | 3 (20.0) | 16 (39.0) |  |
| 5-64 | 12 (21.4) | 4 (26.7) | 8 (19.5) |  |
| 65+ | 2 (3.6) | 2 (13.3) | - |  |
| *Gender* |  |  |  | .07 |
| Male | 43 (76.8) | 9 (60.0) | 34 (82.9) |  |
| Female | 13 (23.2) | 6 (40.0) | 7 (17.1) |  |
| *Marital Status* |  |  |  |  |
| Single | 6 (10.7) | 1 (6.7) | 5 (12.2) |  |
| Married/Living with partner | 44 (78.6) | 12 (80.0) | 32 (78.0) |  |
| Separated/Divorced | 5 (8.9) | 1 (6.7) | 4 (9.8) |  |
| Widowed | 1 (1.8) | 1 (6.7) | - |  |
| *Farming type* |  |  |  |  |
| Beef | 10 (17.9) | 2 (13.3) | 8 (19.5) |  |
| Combinable crops | 15 (26.8) | 2 (13.3) | 13 (31.7) |  |
| Dairy | 5 (8.9) | 3 (20.0) | 2 (4.9) |  |
| Pigs | 3 (5.4) | 2 (13.3) | 1 (2.4) |  |
| Potatoes | 3 (5.4) | 1 (6.7) | 2 (4.9) |  |
| Poultry | 1 (1.8) | - | 1 (2.4) |  |
| Sheep | 13 (23.2) | 5 (33.3) | 8 (19.5) |  |
| Mixed farming | 6 (10.7) | - | 6 (14.6) |  |
| *Time spent on the farm per day* |  |  |  | .92 |
| Less than 4 hours | 6 (10.7) | 2 (13.3) | 4 (9.8) |  |
| 4-6 hours | 3 (5.4) | 1 (6.7) | 2 (4.9) |  |
| 6.5-8 hours | 10 (17.9) | 2 (13.3) | 8 (19.5) |  |
| 8.5-10 hours | 15 (26.8) | 3 (20.0) | 12 (29.3) |  |
| 10.5-12 hours | 14 (25.0) | 4 (26.7) | 10 (24.4) |  |
| 12+ hours | 8 (14.3) | 3 (20.0) | 5 (12.2) |  |
| *Past Mental Health Problem* |  |  |  | .92 |
| Yes | 33 (58.9) | 9 (60.0) | 24 (58.5) |  |
| No | 23 (41.1) | 6 (40.0) | 17 (41.5) |  |
| *Currently on medication* |  |  |  | .31 |
| Yes | 14 (25.0) | 2 (13.3) | 12 (29.3) |  |
| No | 42 (75.0) | 13 (86.7) | 29 (70.7) |  |
| *Experience using the internet* |  |  |  |  |
| 1-3 years | 2 (3.6) | - | 2 (4.9) |  |
| 4-6 years | 7 (12.5) | 3 (20.0) | 4 (9.8) |  |
| 7+ years | 47 (83.9) | 12 (80.0) | 35 (85.4) |  |
| *Continuous Variables* | *Median (IQR)* | *Median (IQR)* | *Median (IQR)* |  |
| *PHQ-9* | 7 (4 – 11) | 8 (4 - 12) | 7 (4 - 11) | .62 |
|  |  |  |  |  |
| *GAD-7* | 6 (2 – 9) | 6 (2 - 13) | 6 (3 - 9) | .52 |
|  |  |  |  |  |
| *WSAS* | 9 (4 – 12) | 9 (4 - 14) | 12 (10 - 20) | .19 |
| ^a^Fisher’s-exact test used for cell counts <5; no test of significance for variables with cell counts <1 | | | | |
