# Supplementary material for: Computerized Cognitive Behavioral Therapy for Anxiety and Depression in Farming Communities: Mixed Methods Feasibility Study of Participant Use and Acceptability
Source: JMIR Form Res. 2023 Jun 19;7:e42573. doi: 10.2196/42573 (PMC10337352; doi:10.2196/42573)
Supplement: Multimedia Appendix 3 [file formative_v7i1e42573_app3.docx]

*Supplementary File 3. Sensitivity analysis for change in measures over time for participants with PHQ-9 ≥ 5*

| *Table 2*. Change in secondary outcome measures over time for participants with PHQ-9 ≥ 5 | | | |
| --- | --- | --- | --- |
|  | *Baseline*  *Median (IQR)* | *Follow-up*  *Median (IQR)* | *Significance*  *P-value* |
| PHQ-9 | 8 (6-14) | 8 (5-9) | .13 |
|  |  |  |  |
| GAD-7 | 9 (5-14) | 9 (4-11) | .04 |
|  |  |  |  |
| WSAS | 11 (9-16) | 10 (7-13) | .41 |
